# Clinical characteristics and long-term progression of young patients with acute coronary syndrome in Brazil

**DOI:** 10.1590/S1679-45082015AO3381

**Published:** 2015

**Authors:** Alexandre de Matos Soeiro, Felipe Lourenço Fernandes, Maria Carolina Feres de Almeida Soeiro, Carlos Vicente Serrano, Múcio Tavares de Oliveira

**Affiliations:** 1 Hospital Israelita Albert Einstein, São Paulo, SP, Brazil.; 2 Instituto do Coração, Hospital das Clínicas, Faculdade de Medicina, Universidade de São Paulo, São Paulo, SP, Brazil.

**Keywords:** Adult, Acute coronary syndrome, Myocardial infarction, Brazil

## Abstract

**Objective:**

In Brazil, there are few descriptions in the literature on the angiographic pattern and clinical characteristics of young patients with acute coronary syndrome, despite the evident number of cases in the population. The objective of this study was to evaluate which clinical characteristics are most closely related to the acute coronary syndrome in young patients, and what long-term outcomes are in this population.

**Methods:**

This is a prospective observational study with 268 patients aged under 55 years with acute coronary syndrome, carried out between May 2010 and May 2013. Data were obtained on demographics, laboratory test and angiography results, and the coronary treatment adopted. Statistical analysis was presented as percentages and absolute values.

**Results:**

Approximately 57% were men and the median age was 50 years (30 to 55). The main risk factors were arterial hypertension (68%), smoking (67%), and dyslipidemia (43%). Typical pain was present in 90% of patients. In young individuals, 25.7% showed ST segment elevation. Approximately 56.5% of patients presented with a single-vessel angiographic pattern. About 7.1% were submitted to coronary bypass surgery, and 42.1% to percutaneous coronary angioplasty. Intrahospital mortality was 1.5%, and the combined event rate (cerebrovascular accident/stroke, cardiogenic shock, reinfarction, and arrhythmias) was 13.8%. After a mean follow-up of 10 months, mortality was 9.8%, while 25.4% of the patients had new ischemic events, and 37.3% required readmission to hospital.

**Conclusion:**

In the short-term, young patients presented with mortality rates below what was expected when compared to the rates noted in other studies. However, there was a significant increase in the number of events in the 10-month follow-up.

## INTRODUCTION

Acute coronary syndrome (ACS) is the primary cause of hospital admission and mortality in the world. In Brazil, however, there are no precise data regarding this in the general population. With increased survival and aging of the world’s population, the ACS is becoming increasingly more frequent. However, not only elderly patients suffer from this disease. Young patients frequently present with unstable angina (UA) episodes and acute myocardial infarction (AMI).^1-3^ Recent changes in lifestyle, eating habits, and work-related stress seem to have an influence and make it appear earlier. Nevertheless, these suppositions have not yet shown scientific confirmation and remain under investigation.^1-3^

The studies with the young population are controversial and rare, and are basically case series and especially, observational studies in the United States, Europe, and Asia.^1-3^ While some authors mention a high prevalence of smoking and familial history of early coronary artery disease as the main risk factors in this population, others show that dyslipidemia is the most prominent factor.^1-3^ Additionally, mortality is extremely variable, and is reported as being between zero and 24% in different series studied.^1-4^

## OBJECTIVE

To evaluate which clinical characteristics are most associated with acute coronary syndrome in young patients, and what the long-term outcomes are in this population.

## METHODS

This is a prospective and observational study. It included all patients aged ≤55 years, diagnosed with ACS (UA and AMI) between May 2010 and May 2013, with a total number of 268 cases (27.8% of the total number of patients with ACS seen during the period evaluated). ACS is considered in all patients who met the criteria established by the latest American Heart Association (AHA) guideline*.* ^4,5^ ACS with ST elevation was defined as the presence of chest pain with persistent alteration of the ST segment ST ≥0.1mV for the frontal plane leads, and ≥0.2mV for the precordial leads, in at least two contiguous leads. ACS with no ST segment elevation was defined as the presence of chest pain associated with electrocardiographic alterations and/or rise/drop of troponin upon admission, or, in the absence of these factors, a clinical picture and risk factors consistent with UA (chest pain when resting or with minimal effort, severe pain, or with a improving pattern). Major bleeding was defined by the score of the Bleeding Academic Research Consortium (BARC)^6^ types 3 and 5, and minor bleeding by types 1 and 2. Reinfarction was considered when there was recurrence of chest pain associated with a new troponin elevation. Ischemic stroke was considered when the patient presented with a new focal motor *déficit* confirmed by computed tomography of the skull. There were no exclusion criteria.

The study was submitted to and approved by the Ethics and Research Committee, under protocol number 689.679, CAAE: 31993014.1.0000.0068. The Informed Consent Form was completed by all the patients enrolled in the study.

The following data were gathered: age, sex, *diabetes mellitus*, systemic arterial hypertension, smoking, dyslipidemia, family history of early coronary artery disease, prior coronary disease (stable angina, prior AMI, prior percutaneous coronary intervention, and prior myocardial revascularization), clinical manifestations, electrocardiographic characteristics, hemoglobin, creatinine, lipid profile, troponin peak, left ventricular ejection fraction, medication used in the first 24 hours of hospitalization, use of illicit drugs, known thrombophilias, coronary anatomy, and coronary treatment adopted (Tables 1 and 2).

**Table 1:** Clinical characteristics of the population involved in the study

Characteristics	
Age (median)	50 (30-55)
Males, %	57
BMI	27.4 (19.3-41.8)
*Diabetes mellitus,* %	25
SAH, %	68
Smoking, %	67
Positive FH for CAD, %	15
Dyslipidemia, %	43
Use of illicit drugs, %	3
Thrombophilias, %	2
Stable angina, %	13
Heart failure, %	3
Prior AMI, %	27
Prior CABG, %	10
Prior PTCA, %	21
Hemoglobin, g/dL	14.35±1.91
Creatinine, mg/dL	1.35±1.54
Troponin (peak), ng/dL	13.70±51.58
LVEF, %	42.73±23.46
ASA, %	95
Betablocker, %	71
Enoxaparin, %	77
Clopidogrel, %	59
ACEI, %	60
Statin, %	83

BMI: body mass index; SAH: systemic arterial hypertension; FH: family history; CAD: coronary artery disease; AMI: acute myocardial infarct; CABG: myocardial revascularization; PTCA: percutaneous coronary angioplasty; LVEF: left ventricle ejection fraction; ASA: acetylsalicylic acid; ACEI: angiotensin-converting enzyme inhibitor.

**Table 2:** Lipid profile of the population studied

Total cholesterol (mean - mg/dL)	175.6
HDL (mean - mg/dL)	35.2
% <40mg/dL (%)	75
% >40mg/dL (%)	25
LDL (mean - mg/dL)	113.8
% >160mg/dL (%)	16.4
% >130mg/dL (%)	34.3
% >100mg/dL (%)	52.2
Triglycerides (mean - mg/dL)	146.4
% <150mg/dL (%)	68.3
% >150mg/dL (%)	31.7

HDL: high-density lipoprotein; LDL: low-density lipoprotein.

Statistical analysis for this study is eminently descriptive. The numerical variables with normal distributions are shown as means and standard deviation; the numerical variables with asymmetric distribution are shown as medians and interquartile variation; whereas the categorical variables are shown as absolute and relative frequencies.

Intra-hospital outcomes evaluated were reinfarction, ischemic stroke, heart failure (Killip III/IV), bleeding (major and/or minor), ventricular arrhythmias (ventricular fibrillation and/or tachycardia), mortality due to all causes, and combined events (reinfarction, ischemic stroke, Killip III/IV, bleeding, ventricular arrhythmias, and mortality).

In the long run, the patients were accompanied for a mean period of 10 months by telephone contact and review of electronic medical records. A total of 164 patients (61% of the initial sample) remained in the evaluation. The outcomes described during this period were reinfarction, progression to heart failure, mortality by all causes, and combined events (reinfarction, heart failure, and mortality).

## RESULTS

The mean age of the patients was 50 years, with a predominance of male patients (57%). The most prevalent risk factors were hypertension (68%), smoking (67%), and dyslipidemia (43%). As to the lipid profile, 75% of the patients had high-density lipoprotein cholesterol (HDL-c) <40mg/dL and only 16.4% had low-density lipoprotein cholesterol (LDL-c) >160mg/dL (Table 2). Baseline characteristics of the population are shown on tables 1 and 2.

**Figure 1:**
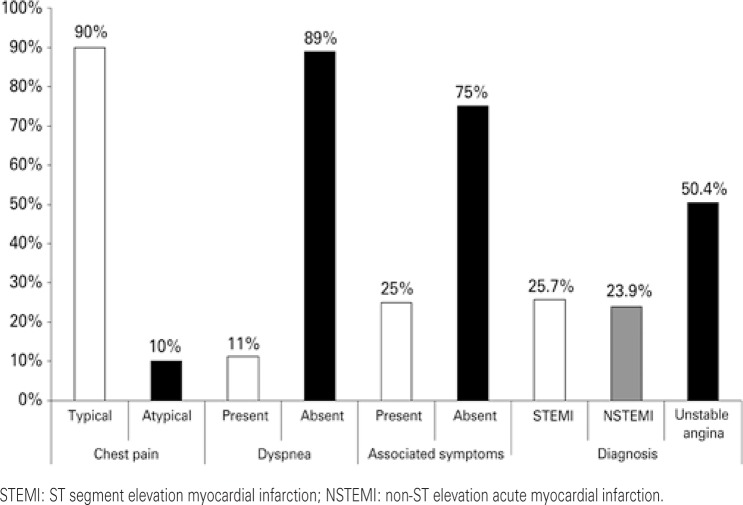
Clinical characteristics and definitive diagnosis in young patients with acute coronary syndrome

Most patients presented with typical pain (90%), with the presence of associated symptoms (sweating and nausea/vomiting) in only 25%. In young individuals, ACS with ST segment elevation was noted in 25.7% (n=69) of the cases. The findings related to clinical presentation are shown on figure 1.

**Figure 2:**
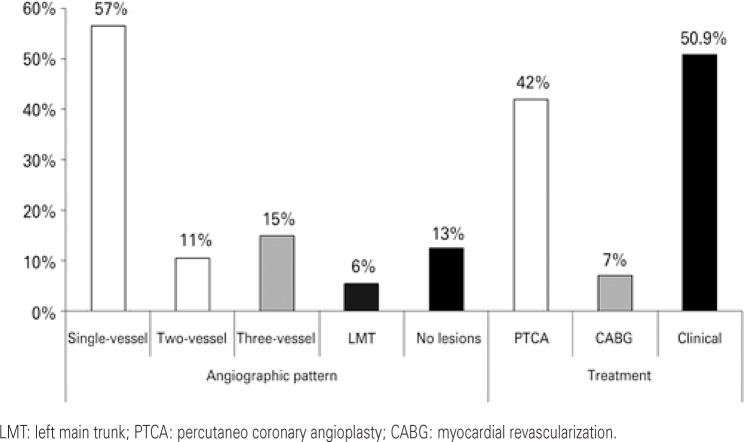
Angiographic characteristics and findings related to coronary treatment in young patients with acute coronary syndrome

In 12.5% of cases, no obstructive coronary lesions were observed (>70% coronary occlusion). In the remaining, single-vessel lesion was seen in 56.5% of patients, two-vessel lesion in 10.5%; three-vessel lesion in 15.0%, and left main trunk lesion in 5.5%. Approximately 7.1% were submitted to myocardial revascularization and 42.1% to coronary angioplasty. The results related to the findings and to the coronary treatment adopted are displayed in figure 2.

Table 3 shows data regarding the in-hospital outcomes. In-hospital mortality found was 1.5%, and the total of combined outcomes was 13.8%.

**Table 3:** In-hospital outcomes in young patients with acute coronary syndrome

Outcomes	%
Reinfarction	0.4
Killip III/IV	3.7
VF/ VT	1.5
Bleeding	6.7
Ischemic stroke	0.0
Mortality	1.5
Combined events	13.8

VF: ventricular fibrillation; VT: ventricular tachycardia.

In the long run, mortality was 9.8%, while 25.4% of the patients presented with new ischemic events, as shown on table 4.

**Table 4:** Long-term outcomes observed in young patients with acute coronary syndrome

Outcomes	%
Reinfarction	25.4
Heart failure	6.7
Mortality	9.8
Combined events	41.9

## DISCUSSION

The first great difficulty found in scientific literature as to ACS in young patients is the definition of which age should be used as cutoff for evaluation, which can vary between 30 and 55 years. The most accepted definition is based on a cutoff point of 55 years, and therefore, it was adopted for this study. Patients aged under 30 years may account for about 0.4% of the general population admitted due to ACS.^7^ In other surveys, patients aged under 40 years accounted for up to 10% of cases, and when under 55 years of age, up to 20% of the total number of indviduals with ACS that seek medical care.^1,2,8,9^ This lack of uniformity as to the definition for age for ACS diagnosis in young patients, along with the low number of articles published on the topic, make the comparison among studies an arduous task. In Brazil, to date, there is no similar evaluation as to the population of young patients with ACS. The finding that approximately 28% of patients seen with ACS are under 55 years of age becomes relevant in this context.

When we evaluated the sex of patients, we observed an absolute predominance of males in literature. This number can vary from 80 to 100% for the 35-45-year age group.^1-3,7,9^ Despite the fact of the population of our study also having been formed primarily by men, in our survey this number was limited to 57% of population. This value is relatively lower when compared with other studies and can be explained by the fact that we did not use such a low cutoff point for age. With increasing age, there is a tendency for the incidence of ACS to be equal in both sexes.

The pathophysiology of ACS in young patients might be different from that found in aged patients. The classic theory of rupture of the atherosclerosis plaque with activation of the coagulation cascade and vessel obstruction was considered rare in the first decades of life until recently. On the other hand, smoking, vessel spasm, thrombophilia, and the use of cocaine seem to be more related to this phase of life.^7,8^ The incidence of thrombophilias and vasospasms reaches up to 76% of patients under the age of 30 years with a diagnosis of AMI.^1^ Despite these descriptions, in the population we studied we only found eight (3%) cases of ACS associated with the use of cocaine, and four (1.5%) cases of thrombophilias (three related to the presence of the antiphospholipid antibody and one to Leiden factor V).

According to Colkesen et al.,^1^ 83% of the population with STEMI aged under 35 years of age are males, and 62% have a history of smoking. Additionally, the population of young patients with STEMI has more family history of early coronary disease and presents with lower HDL-c levels when compared to the population of the same age without cardiovascular disease.^1-3^ In other studies, the percentage of patients who smoke can reach 81% of those with an AMI.^3^ In this way, as is the case in other studies, the percentage of smokers found in this study was high (67%), and this was one of the primary risk factors associated with ACS in young adults. Regarding family history of early coronary disease, Colkesen et al.^1^ described a prevalence of 33% *versus* 16%, when comparing patients with ACS under or over the age of 35 years, respectively.^1^ An interesting fact in our study was the high rate of prior AMI, which was not previously expected in this age range evaluated, but can be explained due to the complexity of the related cardiology care center.

The incidence of *diabetes mellitus* was not very high in the young population. The studies that compared ACS in patients aged under 40 years with those over 40 years demonstrate the incidence of diabetes to be greater in older patients.^8^ This might be explained by the pathophysiology of hyperglycemia in the atherosclerotic process. This process is time-dependent meaning that patients with *diabetes mellitus* with coronary disease might require various decades of life to manifest the problem. On the other hand, as has already been previously described, the pathophysiology of ACS in the young person does not seem to share the same classic risk factors fully.

As to coronary involvement, most of the population described by other authors presented with a single-vessel lesion (69%), which for 60% was the anterior descending artery, while only 14% had a significant three-vessel lesion.^1^ Such findings coincide with our data, reinforcing the relevant presence of single coronary lesions. In our survey, only 12.5% of all patients submitted to catheterization had no significant obstructive coronary lesion. In other studies the percentage of patients with no coronary lesion or with non-obstructive lesions (<50% occlusion) has been noted in up to half the patients with AMI.^7^ In a Japanese study done with patients under the age of 40 years, the result found was closer to that observed in our study, with only one patient of the 27 (3.8%) who had no coronary lesion.^2^

Only a minority of patients was treated surgically. These findings coincide with the majority of descriptions previously presented.^1,2^ However, most patients we described were under clinical treatment, while in other studies, the majority was submitted to percutaneous coronary angioplasty.^1,2^ Maybe this difference is due to the fact of we selected UA patients, with 12.5% of coronary angiographies showing no lesions, whereas most of the other studies selected only AMI patients.

The prognosis of young patients with ACS is still a controversial theme in medical literature. In general, we can note that young patients present with a better short-term prognosis than older patients, with low mortality rates and combined events. According to a survey done by Shiraishi et al.,^2^ the in-hospital mortality rate of patients under 40 years of age was zero during the period observed. Chen et al.^9^ described a 30-day mortality rate of 4.9% in young patients *versus* 8.6% in the aged. In six months, the mortality found among the young and older patients was 5.5% *versus* 9.7%, respectively.^9^ With a mean follow-up of four years, a single study with prolonged follow-up in young patients with ACS demonstrated 28% of need for revascularization in patients with coronary diseases that manifested as AMI.^1^

The study has a few important limitations. Among them is the fact of a large number of losses in follow-up during the study. Additionally, despite reporting the number of patients with thrombophilias and cocaine users in our population, the research was not actively performed in all cases, which could underestimate such findings. And lastly, the limited number of cases did not allow comparisons for different age groups. Nevertheless, this is the first study that prospectively evaluates the short- and long-term outcomes in the young population with ACS, in a Brazilian tertiary center. Moreover, such data, albeit initial, may serve as guidance in the prevention of risk factors and as an alert for early detection of coronary disease in this population.

## CONCLUSION

Within a short period of time, young patients showed mortality rates lower than what was expected when compared to the indices observed in other studies. Nonetheless, we noted a significant increase in the number of events, in the 10-month follow-up.
