# Supplementary material for: Clinical characteristics and long-term progression of young patients with acute coronary syndrome in Brazil
Source: Einstein (Sao Paulo). 2015 Jul-Sep;13(3):370–5. doi: 10.1590/S1679-45082015AO3381 (PMC4943781; doi:10.1590/S1679-45082015AO3381)
Supplement: Translation

# Translation: Características clínicas e evolução em longo prazo de pacientes jovens com síndrome coronariana aguda no Brasil

**Authors:** Alexandre de Matos Soeiro, Felipe Lourenço Fernandes, Maria Carolina Feres de Almeida Soeiro, Carlos Vicente Serrano, Múcio Tavares de Oliveira

**Affiliations:** 1 Hospital Israelita Albert Einstein, São Paulo, SP, Brasil.; 2 Instituto do Coração, Hospital das Clínicas, Faculdade de Medicina, Universidade de São Paulo, São Paulo, SP, Brasil.

**Keywords:** Adulto, Síndrome coronariana aguda, Infarto do miocárdio, Brasil

## Abstract

**Objetivo:**

No Brasil, há poucas descrições na literatura referentes ao padrão angiográfico e a características clínicas em pacientes jovens com síndrome coronariana aguda, apesar do número evidente de casos nessa população. O objetivo deste trabalho foi avaliar quais as características clínicas que mais se associam com a síndrome coronariana aguda em pacientes jovens e quais os desfechos a longo prazo nessa população.

**Métodos:**

Trata-se de estudo prospectivo observacional com 268 pacientes com menos de 55 anos de idade com síndrome coronariana aguda, realizado entre maio de 2010 e maio de 2013. Foram obtidos dados demográficos, laboratoriais, angiográficos e do tratamento coronário adotado. Análise estatística foi apresentada sob a forma de porcentagens e valores absolutos.

**Resultados:**

Aproximadamente 57% eram homens e a mediana de idade foi de 50 anos (30 a 55). Observou-se como principais fatores de risco hipertensão (68%), tabagismo (67%) e dislipidemia (43%). Dor típica esteve presente em 90% dos pacientes. Nos indivíduos jovens, observou-se supradesnivelamento de ST em 25,7% dos casos. Aproximadamente 56,5% dos pacientes apresentaram padrão angiográfico uniarterial. Cerca de 7,1% foram submetidos à cirurgia de revascularização miocárdica e 42,1% à angioplastia coronária. A mortalidade intra-hospitalar encontrada foi de 1,5%, e o índice de desfechos combinados (acidente vascular cerebral, choque cardiogênico, reinfarto e arritmias) foi de 13,8%. Após seguimento médio de 10 meses, a mortalidade foi de 9,8%, enquanto 25,4% dos pacientes apresentaram novos eventos isquêmicos e 37,3%, necessidade de reinternação.

**Conclusão:**

A curto prazo, pacientes jovens apresentam índices de mortalidade abaixo do esperado, quando comparados os índices observados em outros estudos. No entanto, observou-se aumento importante no número de eventos no seguimento de 10 meses.

## INTRODUÇÃO

A síndrome coronariana aguda (SCA) é a principal causa de internação e mortalidade no mundo. No entanto, no Brasil, não existem dados precisos a esse respeito na população geral. Com o aumento da sobrevida e o envelhecimento da população mundial, a SCA vem se tornando cada vez mais frequente. Todavia, não apenas os pacientes idosos sofrem dessa doença. Pacientes jovens apresentam frequentemente episódios de angina instável (AI) e infarto agudo do miocárdio (IAM).^1-3^ Mudanças recentes no estilo de vida, alimentação e estresse relacionado ao trabalho parecem influenciar e tornar mais precoce sua apresentação. No entanto, essas suposições ainda não apresentam confirmações científicas e permanecem sob investigação.^1-3^

Os estudos com a população jovem são controversos e raros, resumindo-se a séries de casos e, principalmente, estudos observacionais nos Estados Unidos, Europa e Ásia.^1-3^ Enquanto alguns autores citam prevalência elevada de tabagismo e história familiar de doença arterial coronariana precoce como os principais fatores de risco nessa população, outros mostram que a dislipidemia é o fator preponderante.^1-3^ Além disso, a mortalidade é extremamente variável, sendo relatada entre zero e 24% em diferentes séries estudadas.^1-4^

## OBJETIVO

Avaliar quais as características clínicas que mais se associam com a síndrome coronariana aguda em pacientes jovens e quais os desfechos a longo prazo nesta população.

## MÉTODOS

Trata-se de estudo prospectivo e observacional. Incluíram-se todos os pacientes com idade ≤55 anos, com diagnóstico de SCA (AI e IAM) entre maio de 2010 e maio de 2013, totalizando 268 casos (27,8% do total de pacientes com SCA atendidos no período avaliado). Consideraram-se como SCA todos os pacientes que preencheram os critérios estabelecidos pela última diretriz da *American Heart Association *(AHA)*.* ^4,5^ SCA com supradesnivelamento do segmento ST foi definida como presença de dor torácica com alteração persistente do segmento ST ≥0,1mV nas derivações do plano frontal, e ≥0,2mV nas derivações precordiais, em pelo menos duas derivações contíguas. SCA sem supradesnivelamento de ST foi definida como presença de dor torácica associada a alterações eletrocardiográficas ou elevação/queda de troponina na internação ou, na ausência destes, quadro clínico e fatores de risco compatíveis com AI (dor torácica ao repouso ou aos mínimos esforços, severa ou ocorrendo com padrão em crescendo). Sangramento maior foi definido pelo escore *Bleeding Academic Research Consortium* (BARC)^6^ tipos 3 e 5, e sangramento menor, pelos tipos 1 e 2. Reinfarto foi considerado quando houve recorrência da dor torácica associada à nova elevação de troponina. Acidente vascular encefálico isquêmico (AVCi) foi considerado quando o paciente apresentasse novo *déficit* neurológico focal motor confirmado por meio de tomografia computadorizada de crânio. Não houve critérios de exclusão.

O trabalho foi submetido e aprovado pelo comitê de ética e pesquisa sob o número de protocolo 689.679, CAAE: 31993014.1.0000.0068. O Termo de Consentimento Livre e Esclarecido foi preenchido por todos os pacientes incluídos no estudo.

Foram coletados os seguintes dados: idade, sexo, *diabetes mellitus*, hipertensão arterial sistêmica, tabagismo, dislipidemia, história familiar de doença arterial coronária precoce, doença coronária prévia (angina estável, IAM prévio, intervenção coronária percutânea prévia e cirurgia de revascularização miocárdica prévia), manifestações clínicas, características eletrocardiográficas, hemoglobina, creatinina, perfil lipídico, pico de troponina, fração de ejeção do ventrículo esquerdo, medicação usada nas primeiras 24 horas de internação, uso de drogas ilícitas, trombofilias conhecidas, anatomia coronária e tratamento coronária adotado (Tabelas 1 e 2).

**Tabela 1:** Características clínicas da população envolvida no estudo

Características	
Idade (mediana)	50 (30-55)
Sexo masculino, %	57
IMC	27,4 (19,3-41,8)
*Diabetes mellitus,* %	25
HAS, %	68
Tabagismo, %	67
HF positiva para DAC, %	15
Dislipidemia, %	43
Uso de drogas ilícitas, %	3
Trombofilias, %	2
Angina estável, %	13
Insuficiência cardíaca, %	3
IAM prévio, %	27
CRM prévia, %	10
ATC prévia, %	21
Hemoglobina, g/dL	14,35±1,91
Creatinina, mg/dL	1,35±1,54
Troponina (pico), ng/dL	13,70±51,58
FEVE, %	42,73±23,46
AAS, %	95
Betabloqueador, %	71
Enoxaparina, %	77
Clopidogrel, %	59
IECA, %	60
Estatina, %	83

IMC: índice de massa corporal; HAS: hipertensão arterial sistêmica; HF: história familiar; DAC: doença arterial coronária; IAM: infarto agudo do miocárdio; CRM: cirurgia de revascularização miocárdica; ATC: angioplastia transluminal coronária; FEVE: fração de ejeção do ventrículo esquerdo; AAS: ácido acetilsalicílico; IECA: inibidor da enzima conversora da angiotensina.

**Tabela 2:** Perfil lipídico da população estudada

Colesterol total (média - mg/dL)	175,6
HDL (média - mg/dL)	35,2
% <40mg/dL (%)	75
% >40mg/dL (%)	25
LDL (média - mg/dL)	113,8
% >160mg/dL (%)	16,4
% >130mg/dL (%)	34,3
% >100mg/dL (%)	52,2
Triglicérides (média - mg/dL)	146,4
% <150mg/dL (%)	68,3
% >150mg/dL (%)	31,7

HDL: lipoproteína de alta densidade; LDL: lipoproteína de baixa densidade.

A análise estatística para esse estudo é eminentemente descritiva. As variáveis numéricas com distribuição normal são apresentadas na forma de média e desvio padrão; as variáveis numéricas com distribuição assimétrica são apresentadas na forma de mediana e variação interquartil; enquanto as variáveis categóricas são apresentadas na forma de frequências absolutas e relativas.

Os desfechos intrahospitalares avaliados foram reinfarto, AVCi, insuficiência cardíaca (Killip III/IV), sangramento (maior e/ou menor), arritmias ventriculares (fibrilação e/ou taquicardia ventricular), mortalidade por todas as causas e eventos combinados (reinfarto, AVCi, Killip III/IV, sangramento, arritmias ventriculares e mortalidade).

A longo prazo, os pacientes foram acompanhados por um período médio de 10 meses por meio de contato telefônico e revisão de prontuário eletrônico. Permaneceram nessa avaliação 164 pacientes (61% da amostra inicial). Os desfechos descritos nesse período foram reinfarto, evolução para insuficiência cardíaca, mortalidade por todas as causas e eventos combinados (reinfarto, insuficiência cardíaca e mortalidade).

## RESULTADOS

A média da idade dos pacientes foi de 50 anos, com predomínio de pacientes do sexo masculino (57%). Os fatores de risco mais prevalentes foram hipertensão (68%), tabagismo (67%) e dislipidemia (43%). Em relação ao perfil lipídico, 75% dos pacientes tinham lipoproteína de alta densidade-colesterol (HDL-c) <40mg/dL e apenas 16,4% tinham lipoproteína de baixa densidade-colesterol (LDL-c) >160mg/dL (Tabela 2). As características basais da população encontram-se nas tabelas 1 e 2.

A maioria dos pacientes apresentou dor considerada típica (90%), com presença de sintomas associados (sudorese e náuseas/vômitos) em apenas 25%. Em indivíduos jovens, observou-se SCA com supradesnivelamento de ST em 25,7% (n=69) do total de casos. Os achados relacionados à apresentação clínica encontram-se na figura 1.

**Figura 1:** Características clínicas e diagnóstico definitivo em pacientes jovens com síndrome coronariana aguda

Em 12,5% dos casos, não foram observadas lesões coronárias obstrutivas (>70% de oclusão coronária). No restante, observou-se lesão uniarterial em 56,5% dos pacientes, 10,5% biarterial, 15,0% triarterial e 5,5% com lesão de tronco de coronária esquerda. Cerca de 7,1% foram submetidos à cirurgia de revascularização miocárdica e 42,1% à angioplastia coronária. Os resultados referentes aos achados e ao tratamento coronário adotado podem ser observados na figura 2.

**Figura 2:** Características angiográficas e relacionadas ao tratamento coronário em pacientes jovens com síndrome coronariana aguda

A tabela 3 mostra dados referentes aos desfechos intra-hospitalares. A mortalidade intra-hospitalar encontrada foi de 1,5% e o total de desfechos combinados foi de 13,8%.

**Tabela 3:** Desfechos intra-hospitalares em pacientes jovens com síndrome coronariana aguda

Desfechos	%
Reinfarto	0,4
Killip III/IV	3,7
FV/TV	1,5
Sangramento	6,7
AVCi	0,0
Mortalidade	1,5
Eventos combinados	13,8

FV: fibrilação ventricular; TV: taquicardia ventricular; AVCi: acidente vascular cerebral isquêmico.

A longo prazo, a mortalidade foi de 9,8%, enquanto 25,4% dos pacientes apresentaram novos eventos isquêmicos, conforme apresentado na tabela 4.

**Tabela 4:** Desfechos observados em longo prazo em pacientes jovens com síndrome coronariana aguda

Desfechos	%
Reinfarto	25,4
Insuficiência cardíaca	6,7
Mortalidade	9,8
Eventos combinados	41,9

## DISCUSSÃO

A primeira grande dificuldade encontrada na literatura científica em relação à SCA em pacientes jovens é a definição de qual idade deve ser utilizada como corte para avaliação, podendo variar de 30 a 55 anos. A definição mais aceita baseia-se no corte de 55 anos e, por isso, foi a adotada neste trabalho. Pacientes com menos de 30 anos podem representar cerca de 0,4% da população geral admitida por SCA.^7^ Em outros levantamentos, pacientes com menos de 40 anos podem perfazer até 10% e, com menos de 55 anos, até 20% do total com SCA que procuram atendimento médico.^1,2,8,9^ Essa falta de uniformidade em relação à definição da idade para diagnóstico de SCA em pacientes jovens, juntamente do baixo número de artigos publicados a esse respeito, faz com que a comparação entre estudos seja uma tarefa árdua. No Brasil, não existe, até o momento, uma avaliação semelhante em relação à população de pacientes jovens com SCA. O achado de aproximadamente 28% dos pacientes atendidos com SCA possuírem menos de 55 anos torna-se algo relevante nesse contexto.

Quando avaliamos o sexo dos pacientes, verificamos um predomínio absoluto do sexo masculino na literatura. Esse número pode variar de 80 a 100% quando nas faixas etárias de 35 a 45 anos.^1-3,7,9^ Apesar da população do nosso estudo também ser formada principalmente por homens, no nosso levantamento esse número se limitou a 57% da população. Esse valor é relativamente inferior quando comparado com outros estudos e pode ser explicado pelo fato de não termos utilizado um corte de idade tão baixo. Com o aumento da idade, existe a tendência de que a incidência de SCA se iguale em ambos os sexos.

A fisiopatologia da SCA em pacientes jovens pode ser diferente daquela encontrada em paciente idosos. A clássica teoria de ruptura da placa de aterosclerose com ativação da cascata de coagulação e obstrução do vaso era considerada rara nas primeiras décadas de vida há até pouco tempo. Por outro lado, o tabagismo, o vasoespasmo, as trombofilias e o uso de cocaína parecem estar mais relacionados nessa fase da vida.^7,8^ A incidência de trombofilias e vasoespasmo chega a representar cerca de 76% dos pacientes com menos de 30 anos com diagnóstico IAM.^1^ Apesar dessas descrições, somente encontramos na população estudada oito (3%) casos de SCA associadas ao uso de cocaína e quatro (1,5%) casos de trombofilias (três relacionados à presença de anticorpo antifosfolípide e um ao fator V de Leiden).

De acordo com Colkesen et al.,^1^ 83% da população com IAM com supradesnivelamento de ST abaixo dos 35 anos pertencem ao sexo masculino e 62% têm histórico de tabagismo. Além disso, a população de pacientes jovens com IAM com supradesnivelamento de ST tem maior histórico familiar de doença coronária precoce e apresenta menores níveis de HDL-c quando comparada à população da mesma idade sem doença cardiovascular.^1-3^ Em outros estudos, a porcentagem de pacientes tabagistas pode checar a 81% daqueles com IAM.^3^ Dessa forma, assim como em outros estudos, a porcentagem de tabagismo encontrada nesse estudo foi elevada (67%), sendo este um dos principais fatores de risco associados à SCA em jovens. Já em relação à história familiar de doença coronária precoce, Colkesen et al.^1^ descreveram uma prevalência de 33% contra 16%, ao comparar pacientes com SCA com menos ou mais de 35 anos de idade, respectivamente.^1^ Dado interessante em nosso estudo foi a taxa elevada de IAM prévio, algo previamente não esperado nessa faixa etária avaliada, mas que pode ser explicado devido à complexidade do centro de atendimento cardiológico relacionado.

A incidência de *diabetes mellitus *não foi muito elevada na população jovem. Os trabalhos que compararam SCA em pacientes com menos que 40 àqueles acima de 40 anos demonstram a incidência de diabetes maior nos pacientes mais idosos.^8^ Isso pode ser explicado pela fisiopatologia da hiperglicemia no processo de aterosclerose. Esse processo é tempo dependente, de tal forma que os pacientes com *diabetes mellitus* podem demorar várias décadas para manifestar a doença coronariana. Por outro lado, conforme já descrito anteriormente, a fisiopatologia de SCA no jovem não parece compartilhar por completo os mesmos fatores de risco clássicos.

Em relação ao acometimento coronário, a maioria da população apresentada por outros autores apresentava lesão univascular (69%), sendo que, em 60% das vezes, a artéria culpada é a descendente anterior, enquanto apenas 14% têm lesão triarterial significativa.^1^ Tais achados coincidem com nossos dados, reforçando a presença relevante de lesões coronárias únicas. No nosso levantamento, apenas 12,5% de todos os pacientes submetidos a cateterismo não apresentavam lesão coronariana obstrutiva significativa. Em outros estudos a porcentagem de pacientes sem nenhuma lesão coronariana ou com lesões não obstrutivas (<50% de oclusão) chega a ser observada em até metade dos pacientes com IAM.^7^ Em um estudo japonês em pacientes com menos de 40 anos, o resultado encontrado foi mais parecido com o observado no nosso estudo, sendo que apenas 1 paciente dos 27 (3,8%) não apresentava lesão coronariana.^2^

Apenas uma minoria dos pacientes foi tratada cirurgicamente. Esses achados coincidem com a maioria das descrições previamente apresentadas.^1,2^ Todavia, a maioria dos pacientes aqui descritos ficou em tratamento clínico, enquanto em outros trabalhos, a maior parte foi submetida à angioplastia coronária percutânea.^1,2^ Talvez tal diferença se deva ao fato de termos selecionados pacientes com quadro de AI, com 12,5% das angiografias coronárias sem lesões, enquanto a maioria dos outros estudos selecionaram apenas pacientes com IAM.

O prognóstico de pacientes jovens com SCA ainda é tema de controvérsia na literatura médica. Em geral, podemos verificar que pacientes mais jovens apresentam melhor prognóstico a curto prazo do que pacientes mais idosos, com baixos índices de mortalidade e eventos combinados. Segundo levantamento de Shiraishi et al.,^2^ a mortalidade intra-hospitalar de pacientes com menos de 40 anos foi zero durante o período observado. Chen et al.^9^ descreveram uma mortalidade em 30 dias de 4,9% em pacientes jovens contra 8,6% nos idosos. Em 6 meses, a mortalidade encontrada em jovens e idosos foi de 5,5% contra 9,7%, respectivamente.^9^ Com seguimento médio de 4 anos, o único estudo com acompanhamento prolongado em pacientes jovens com SCA demonstrou 28% de necessidade de revascularização nos pacientes com coronariopatias que se manifestaram como IAM.^1^

O estudo apresenta algumas limitações importantes. Entre elas, está o fato de um número grande de perda de seguimento durante o estudo. Além disso, apesar de relatado o número de pacientes com trombofilias e usuários de cocaína em nossa população, a pesquisa não foi realizada ativamente em todos os casos, o que pode subestimar tais achados. Por último, a casuística limitada não permitiu comparações em diferentes faixas etárias. Apesar disso, este é o primeiro estudo que avalia de maneira prospectiva os desfechos a curto e a longo prazo na população jovem com SCA em um centro terciário brasileiro. Além disso, tais dados, apesar de iniciais, podem servir como orientadores à prevenção de fatores de risco e como alerta da detecção precoce da doença coronariana nessa população.

## CONCLUSÃO

A curto prazo, pacientes jovens apresentam índices de mortalidade abaixo do esperado, quando comparados a índices observados em outros estudos. No entanto, observou-se aumento importante no número de eventos, no seguimento de 10 meses.
